# Intravascular large B-cell lymphoma presenting as multiple stroke: A case report

**DOI:** 10.1097/MD.0000000000012793

**Published:** 2018-10-12

**Authors:** Muharrem Yunce, Nargiz Muganlinskaya, Stephen Selinger

**Affiliations:** a Department of Medicine; b Department of Pulmonary and Critical Care Medicine, MedStar Franklin Square Medical Center, Baltimore, MD, USA.

**Keywords:** IVLBCL, skin biopsy, stroke

## Abstract

**Introduction:**

Intravascular large B-cell lymphoma (IVLBCL) is an uncommon disease with a poor prognosis if not diagnosed early. It can present with central nervous system (CNS) manifestations. The diagnosis of IVCBCL is difficult to make given its varied clinical manifestations and the lack of a specific diagnostic modality.

**Case presentation:**

We report an interesting case of IVLBCL presenting as bilateral strokes. The diagnosis was made by a random skin biopsy, which confirmed IVLBCL. The patient was treated with rituximab, cyclophosphamide, doxorubicin, vincristine, and prednisone (R-CHOP).

Neurological symptoms improved with R-CHOP. Repeat magnetic resonance imaging (MRI) of the brain showed improvement of the prior lesions.

**Conclusion:**

IVLBCL is an aggressive disease with high mortality. Timely diagnosis and treatment can be lifesaving.

## 1 Introduction

Intravascular large B-cell lymphoma (IVLBCL) is a rare subtype of extra nodal large B-cell lymphoma with an aggressive clinical course. The diagnosis is difficult, as it presents without any obvious tumor mass or lymphadenopathy. This entity was first described by Pfleger and Tappeiner and was called ”angioendotheliomatosis proliferans systemisata.”^[1]^

The incidence of IVLBCL is reported to be less than 1 person per million. The median age at presentation is 70, ranging from 34 to 90 years. Men and women appear to be equally affected.^[2]^ Patients most commonly present with B symptoms, anemia, and a high serum lactate dehydrogenase (LDH) level. The brain and skin are the most commonly involved organs. Involvement of the liver, spleen, and bone marrow has been reported less commonly.^[3]^ Our case was diagnosed with IVLBCL after being hospitalized multiple times due to persistent neurological symptoms.

The clinical picture we report is unique. Complex features precluded a timely diagnosis for this patient, resulting in multiple hospitalizations over 10 months. The patient's symptoms progressed despite treatment of a presumed stroke, panic attacks, conversion disorder, and Hashimoto's encephalopathy. The diagnosis was made with a random skin biopsy, culminating in successful treatment.

## 2 Case presentation

The patient was a 63 year-old Caucasian woman whose history was significant for stroke, depression, and panic attacks. She developed multiple neurological symptoms, cognitive decline, and personality changes.

The patient initially presented with repetitive right hand and arm numbness which recurred approximately every 2 weeks with an average duration of 2 to 5 minutes. While shopping, she suddenly developed a left sided facial droop and vertigo which were self-limited and resolved by the following day. Two additional episodes occurred within the next 1 month. The patient started to experience numbness in other locations. When these episodes escalated, she presented for further evaluation.

During her first hospitalization, the patient underwent magnetic resonance imaging (MRI) of the brain. This demonstrated an old left cerebellar lacunar infarction. Her symptoms were attributed to a stroke; aspirin and a statin were initiated. She was also found to have hypothyroidism which was treated with levothyroxine.

The patient subsequently presented with similar neurological symptoms 2 weeks after discharge. A computerized tomography (CT) of her head showed no interval changes. Since she was reporting significant stressors in her life there was a concern that her symptoms were due to panic attacks. She was started on alprazolam.

Two months after her second discharge, the patient started to experience episodes of confusion and bilateral lower extremity weakness without bowel or bladder incontinence. A cane was required for ambulation. Her weakness was attributed to statin therapy. Her symptoms did not improve despite cessation of her statin, requiring repeat hospitalization. During that hospitalization, an MRI of the lumbar and thoracic spine did not show evidence of spinal cord compression. An electromyogram (EMG) and nerve conduction studies (NCS) were not suggestive of peripheral neuropathy or myopathy. The patient was discharged with a diagnosis of conversion disorder. Outpatient psychiatry and psychology follow up were arranged.

Three months later, the patient was again hospitalized with additional symptoms. She had stopped paying her bills, and started to experience irritability. She became bed confined due to lower extremity weakness. At the time of presentation, her vital signs were normal. The neurological exam was remarkable for confabulation and impaired repetition. The patient was oriented only to her name. Her strength was 3/5 in both lower extremities.

**Figure 1:**
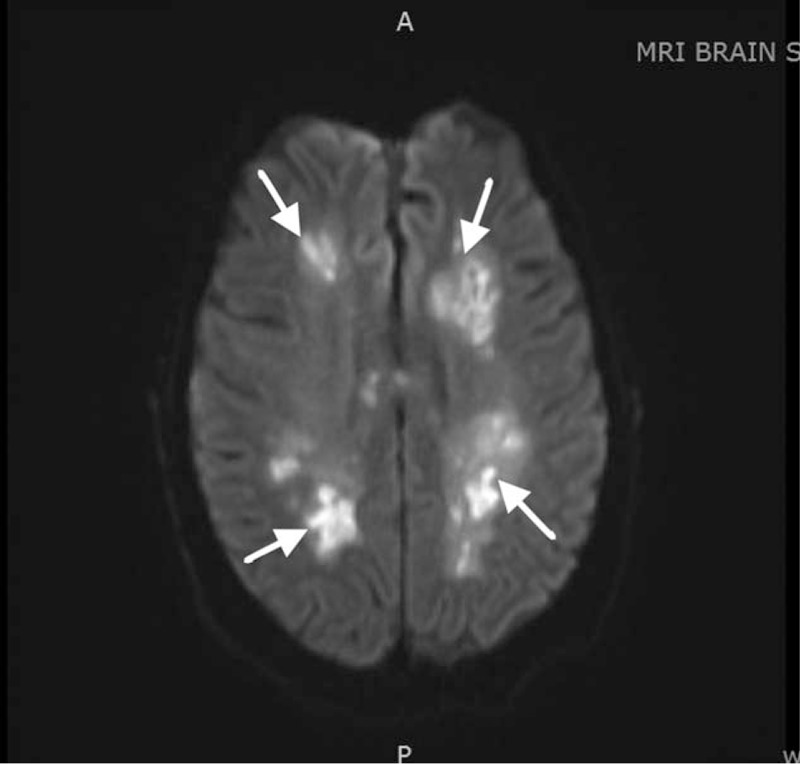
Axial MRI image before treatment demonstrates multifocal bilateral white matter lesions. MRI = magnetic resonance imaging.

A CT of her head showed hypoattenuation in the periventricular white matter. Routine CSF analysis was within normal limits. An electroencephalography (EEG) showed temporal epileptiform discharges bilaterally. An MRI of the brain showed bilateral multifocal white matter changes (Fig. 1). A transthoracic echocardiogram showed an ejection fraction of 65% with no wall motion abnormalities. The thyroglobulin level was >1000 IU/mL.

The patient's symptoms were thought to be consistent with Hashimoto's encephalopathy and seizures. Treatment with IV steroids and levatiracetam was initiated.

The patient's symptoms did not improve with steroids. Given the bilateral white matter changes and rapid progression of disease, an extensive investigation was undertaken. The patient was evaluated for autoimmune disorders, encephalitides (viral, arthropod-borne, autoimmune, and paraneoplastic), neurodegenerative disorders, neoplasms, vasculitides, and inherited angiopathy.

The patient was anemic (hemoglobin 7.0 g/dL) and thrombocytopenic (platelet 80,000 per microliter) with an elevated LDH of 527 U/L. Syphilis and human immunodeficiency virus (HIV) testing were negative. A bone marrow biopsy was unremarkable. A T cell gene rearrangement flow cytometry was normal.

Anti-nuclear antibodies, Rheumatoid factor, Anti-double-stranded DNA, Anti-Ro/SSA, Anti-La/SSB, Anti Jo-1, Anti-proteinase-3, Anti-myeloperoxidase, Anti-Sm, and Anti-RNP antibodies were negative. C3 and C4 levels were negative.

Cerebrospinal fluid studies, including oligoclonal bands, major basic protein, herpes simplex virus-1 and 2 DNA, Epstein-Barr virus DNA, and cryptococcal antigen were negative.

Evaluation for arthropod-borne encephalitides including West Nile virus, Eastern equine encephalitis virus, St. Louis encephalitis virus, Enterovirus, and La Crosse virus was negative. Fungal culture of the CSF was negative.

Workup for neurodegenerative disorders including Tau protein and 14-3-3 protein failed to reveal any abnormality. Notch-3 gene mutation was negative. NMDA receptor antibodies (serum and CSF) were also negative.

A conventional angiogram revealed no evidence of vasculitis. A PET/CT was notable for mild diffuse FDG uptake throughout the visualized axial and appendicular skeleton. She was also noted to have mild to moderate splenomegaly.

Subsequently a random skin biopsy confirmed the presence of IVLBCL.

**Figure 2:**
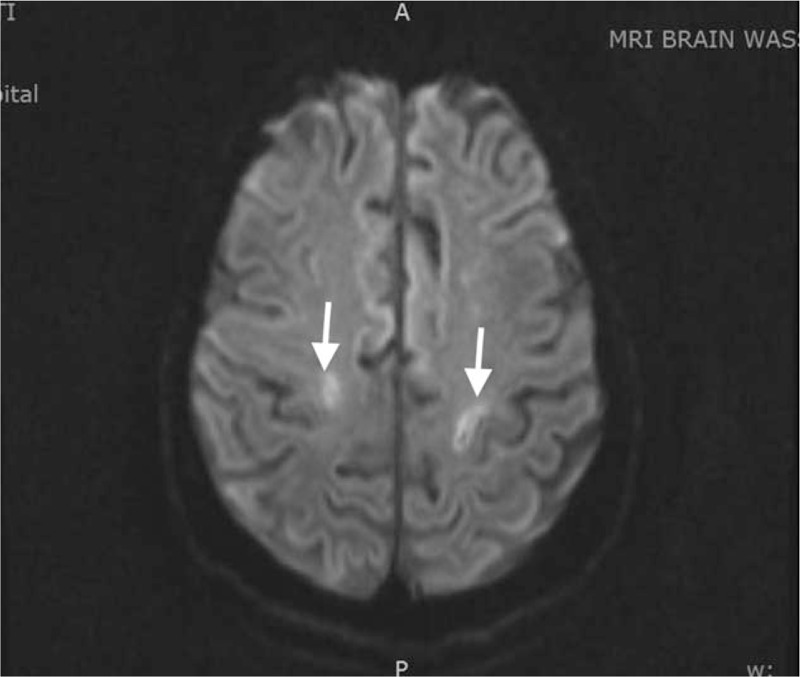
Axial MRI image after chemotherapy. The lesions mostly regressed. MRI = magnetic resonance imaging.

The patient was started on chemotherapy with rituximab, cyclophosphamide, doxorubicin, vincristine, and prednisone (R-CHOP). During a follow-up visit, it was noted that her cognitive function, weakness, writing skills, and speech had all improved. A repeat MRI of her brain showed improvement of the prior lesions (Fig. 2)

## 3 Discussion

The clinical presentation of intravascular lymphoma can resemble multiple different diseases, including demyelinating disease, vasculitis, stroke, as well as rare syndromes such as cerebral autosomal dominant arteriopathy with subcortical infarcts and leukoencephalopathy (CADASIL).^[4–7]^

The diagnosis in our patient was difficult due to the rarity of the disease and the nature of her symptoms. Her presentation was initially attributed to a stroke. A more through history might have suggested a more complex condition, given the episodic nature of her presentation.

There are 2 well described major patterns of IVLBCL. The western variant is characterized by symptoms of multiorgan involvement, most commonly affecting the nervous system or skin. The Asian variant often presents with hemophagocytic syndrome leading to hepatosplenomegaly, pancytopenia, and multiorgan failure.^[3,]^

IVLBCL is a well described entity, but its complex clinical presentation with multi-system involvement and aggressive disease course often challenges physicians to make a timely and accurate diagnosis. Consequently, one-half of patients with IVLBCL are diagnosed post–mortem.^[3]^ An awareness of this disease along with a high index of suspicion can result in a correct diagnosis.

The diagnosis of IVLBCL is usually made by demonstrating excessive proliferation of CD20 positive lymphoma cells within the lumina of small vessels and capillaries. Even though there are case reports with involvement of large arteries and veins, these are mostly spared.^[8]^ Since these lymphoma cells do not circulate, flow cytometry and bone marrow biopsy usually fail to show any abnormality. Random skin biopsy has been shown to be an effective way of making a diagnosis.^[9–11]^ A recent study found open muscle biopsy of high yield in making a diagnosis of IVLBCL.^[12]^

The most common laboratory findings of IVLBCL include anemia, as well as elevated LDH and b-2-microglobulin levels. Thrombocytopenia and leukopenia are less common. The peripheral blood smear, flow cytometry, and genetic studies are usually unremarkable due to a lack of circulating tumor cells as previously mentioned.^[8]^ In our patient, laboratory findings were consistent with previous reports including anemia, thrombocytopenia, and an elevated LDH level.

MRI of the brain is done for most patients who present with cognitive decline. Our patient initially was thought to be a candidate for brain biopsy given her extensive bilateral brain lesions, but fortunately the diagnosis was established with a random skin biopsy. PET/CT imaging can be helpful in some cases but several studies have also suggested significant false negative results.^[13,14]^ In our patient, PET/CT imaging was inconclusive and not helpful.

The treatment for IVLBCL is similar to the regimen used for non-Hodgkins lymphoma. This includes chemo-immunotherapy including rituximab. Ferreri et al investigated the addition of rituximab to the CHOP regimen in patients with IVLBCL. They found significant improvement in complete remission rates, event-free survival, and overall survival.^[15]^

Our patient substantially improved after receiving her first cycle of R-CHOP. On a follow-up visit, she reported improvement of cognitive function, writing skills, lower extremity weakness, and overall health.

In summary, this case illustrates that IVLBCL should remain in the differential diagnosis of patients who have a rapid cognitive decline with multi-organ dysfunction whose laboratory findings demonstrate an elevated LDH and cytopenias. IVLBCL can mimic multiple different diseases given the involvement of multiple small vessels within several organs. Due to the lack of circulatory lymphoma cells, the diagnosis of IVLBCL remains a challenge.

## Author contributions

M. Yunce, completed the background research, drafted and edited the manuscript and the grantor of the publication. N. Muganlinsakaya, S. Selinger edited and completed the manuscript.

**Writing – original draft:** Muharrem Yunce.

**Writing – review & editing:** Muharrem Yunce, Nargiz Muganlinskaya, Stephen Selinger.

Muharrem Yunce orcid: 0000-0002-8634-6410.
